# Genotype-by-environment interactions for starch, mineral, and agronomic traits in pearl millet hybrids evaluated across five locations in West Africa

**DOI:** 10.3389/fpls.2023.1171773

**Published:** 2023-05-23

**Authors:** Prakash I. Gangashetty, Chandra Bhan Yadav, Mohammed Riyazaddin, Anilkumar Vermula, Peter Anabire Asungre, Ignatitius Angarawai, Luis A. J. Mur, Rattan S. Yadav

**Affiliations:** ^1^ Crop Breeding cluster, International Crops Research Institute for the Semi-Arid Tropics, (ICRISAT), Hyderabad, India; ^2^ Crop Breeding cluster, International Crops Research Institute for the Semi-Arid Tropics, (ICRISAT), Niamey, Nigeria; ^3^ Institute of Biological Environmental and Rural Sciences (IBERS), Aberystwyth University, Aberystwyth, United Kingdom; ^4^ Department of Genetics, Genomics, and Breeding, National Institute of Agricultural Botany-East Malling Research Station (NIAB-EMR), East Malling, United Kingdom; ^5^ Millet Breeding, CSIR-Savanna Agriculture Research Institute, Bawku, Ghana; ^6^ Seed Systems cluster, International Crops Research Institute for the Semi-Arid Tropics, (ICRISAT), Kano, Nigeria

**Keywords:** pearl millet, total starch, rapidly digestible starch, slowly digestible starch, resistant starch, genotype x environment interaction, iron, zinc

## Abstract

**Introduction:**

Pearl millet is a staple cereal grown in the harshest environments of arid and semi-arid regions of Asia and sub-Saharan Africa. It is the primary source of calories for millions of people in these regions because it has better adaptation to harsh environmental conditions and better nutritional traits than many other cereals. By screening the pearl millet inbred germplasm association panel (PMiGAP), we earlier reported the best genotypes with the highest concentration of slowly digestible and resistant starch in their grains.

**Methods:**

In the current study, we tested these 20 top-performing pearl millet hybrids, identified based on starch data, in a randomised block design with three replications at five locations in West Africa, viz. Sadore and Konni (Niger), Bambey (Senegal), Kano (Nigeria), and Bawku (Ghana). Phenotypic variability was assessed for agronomic traits and mineral traits (Fe and Zn).

**Results and discussion:**

Analysis of variance demonstrated significant genotypic, environmental, and GEI effects among five testing environments for agronomic traits (days to 50% flowering, panicle length, and grain yield), starch traits (rapidly digestible starch, slowly digestible starch, resistant starch, and total starch), and mineral trait (iron and zinc). Starch traits, such as rapidly digestible starch (RDS) and slowly digestible starch (SDS), showed nonsignificant genotypic and environmental interactions but high heritability, indicating the lower environmental influence on these traits in the genotype × testing environments. Genotype stability and mean performance across all the traits were estimated by calculating the multi-trait stability index (MTSI), which showed that genotypes G3 (ICMX207070), G8 (ICMX207160), and G13 (ICMX207184) were the best performing and most stable among the five test environments.

## 1 Introduction

Pearl millet (*Pennisetum glaucum* L.) is an important cereal crop in Asia and Africa’s arid and semi-arid tropical regions. It is highly resilient to drought and heat and can be cultivated in low-fertility soils, which cause frequent failures of crop production of other cereals (Gupta et al., 2015; Yadav et al., 2015). It is grown on over 30 million hectares, with more than 30.08 million metric tonnes of grain production worldwide (Raheem et al, 2021). It provides the primary source of calories for millions of people in semi-arid tropical areas of Africa and India because of its better adaptation to harsh environmental conditions (Yadav et al., 2002; Bidinger and Hash, 2004; Govindaraj et al., 2010). Approximately 40% of the world’s pearl millet is grown in Africa; about 85% is in West Africa. Africa’s major pearl-millet-producing countries include Nigeria (5 M ha), Niger (7 M ha), Burkina Faso (1.5 M ha), Chad (3.0 M ha), Mali (1.5 M ha), and Senegal (1.0 M ha).

Apart from superior adaptation, pearl millet also possesses a range of health-benefiting traits, such as high grain iron (Fe) and zinc (Zn) content along with starch components that are helpful to type 2 diabetics (Kam et al., 2016). The number of (type 2) mainly noninsulin-dependent diabetics in the African region is increasing. World Health Organisation (WHO) estimates that more than 25 million of Africa’s population was type 2 diabetes (T2D) in 2014, up from 2.6 million in 1980 and projected to be 41 million in 2045. It is estimated that for a low-income family in the African region with a diabetic adult, as much as 25% of family income may be devoted to diabetes care. Apart from high-quality starch, the nutritional value of millets is high as they are rich in nutritionally important minerals like iron, calcium, zinc, magnesium, phosphorous, and potassium (Antony and Chandra, 1998; Vadivoo et al., 1998). In addition, they are also a good source of dietary fibre and several vitamins (β-carotene, niacin, vitamin B6, and folic acid). Insufficient intakes of diet, especially minerals, by humans lead to dysfunctions and diseases in humans and are the primary cause of micronutrient malnutrition in many parts of South Asia and sub-Saharan Africa. Maintenance and improvement of nutrient content in a crop like a pearl millet together with yield are therefore important requirements in pearl millet breeding programs.

The current study focused on testing pearl millet hybrids on parental lines that are known to possess the best combination of starch traits (Yadav et al., 2022). The study’s primary aim was to understand the effects of G×E on starch, yield, and grain nutritional traits and if they can be combined in future varieties. The study also aimed at identifying hybrids possessing the best combination of these traits for cultivation by farmers in these environments.

## 2 Materials and methods

### 2.1 Plant materials

The experimental pearl millet hybrids used in this study comprised 20 lines identified from within the 166 PMiGAP accessions, primarily based on the higher slowly digestible starch (SDS) and resistant starch (RS) content reported by Yadav et al. (2022) in their grains (**Supplementary Table S1**). Two female lines, ICMA17002 and ICMA177111, were used as females for crossing with these lines to produce hybrid seeds during the off-season of 2019 at ICRISAT-Sadore in Niger Republic. Female and male parents used each hybrid combination are presented in **Supplementary Figure S1**.

### 2.2 Experimental trials and field design

Field trials of 20 pearl millet hybrids produced as above were conducted at five locations in West Africa: Bawku–Ghana (E1), Konni–Niger (E2), Kano–Nigeria (E3), Sadore–Niger (E4), and Bambey–Senegal (E5). Geographic coordinates (latitude, longitude, and altitude), type of soil, and weather data during the cropping season (total rainfall, minimum and maximum temperature) for each location are given in **Supplementary Table S2**. An experimental trial was laid out in a randomised complete block design with three replications across five locations. The trial was sown in a two-row plot with a row length of 3 m long and a row-to-row distance of 0.8 m. Thinning was performed in all the locations 15–21 days after sowing. Weeding was carried out when necessary. Recommended agronomic practices were followed in each location.

### 2.3 Agronomic data collections

Phenotypic data for four agronomic traits, namely days to 50% flowering (days), plant height (cm), panicle length (cm), and grain yield (g/plot), were evaluated during the trials, and the data were collected for these traits. The grain yield was computed to tonnes per hectare using primary grain weight data. These traits were measured as per DUS characterisation guidelines. At physiological maturity, each plot was harvested, and samples were collected for grain iron, zinc estimation, and starch component traits. When harvesting and selecting suitable heads, representative grain sampling was done; plants grounded and contaminated with soil were not taken.

### 2.4 Starch content quantification in pearl millet hybrid

Pearl millet grain starch was digested to quantify the rapidly digestible starch (RDS), SDS, and RS fractions (Englyst et al., 1992; Englyst et al., 1999; Yadav et al., 2022). All analyses for starch measurements were performed in duplicate (two replicates) for each hybrid from the milled samples. The samples were milled in a centrifugal miller with a 0.5-mm sieve (Fritsch Pulverisette). After the pretreatment of samples under gastric conditions, the samples were digested using amylolytic enzymes under condition-controlled conditions as described by Yadav et al. (2022). Samples were taken from the digested mix at 20 and 120 min to measure the rate and extent of starch digestion. The release of glucose was estimated by high-pressure ion chromatography (HPIC). Various components of starch, such as RDS, were estimated by measuring the release of glucose in the first 20 min, while SDS content was recorded by measuring the glucose release between 20 and 120 min. Finally, the unhydrolysed starch component after 120 min was considered RS.

### 2.5 Iron and zinc content quantification in pearl millet hybrids

Pearl millet grain micronutrient estimation was carried out using the energy dispersive X-ray fluorescence (EDXRF) method (Paltridge et al., 2012a; Paltridge et al., 2012b; Guild and Stangoulis, 2016). EDXRF analysis was carried out using an Oxford X-Supreme 8000 with a 10-sample carousel. Analyses were conducted in supplied sample cups prepared as reported previously (Paltridge et al., 2012a; Paltridge et al., 2012b; Guild and Stangoulis, 2016), with 4 μm Poly-4 XRF sample film used to seal one end of the cup. Sample cups were cleaned and prepared before each analysis to minimise cross-contamination between samples. Samples of >5 g were used for all analyses to ensure samples were “infinitely thick” in terms of EDXRF analysis (Paltridge et al., 2012b). Calibrations for Fe and Zn in pearl millet were achieved using 20 standards based on inductively coupled plasma‐optical emission spectrophotometry (ICP-OES) data.

### 2.6 Statistical analysis and phenotypic diversity in pearl millet hybrid

A combined analysis of variance across locations was performed using replicated data to test the significance of environment (E), genotype (G), and genotype-by-environment interaction (GEI) using the MIXED procedure of SAS 9.4 (Institute Inc, S. A. S., 2018), considering environment, genotype, and replication as random effects. Individual location variances were modelled in the combined analysis. Predicted means were estimated for E, G, and GEI from combined analysis. Broad sense heritability (*H* ^2^) was estimated for combined analysis by following the formula

$H^{2}=\;\sigma g^{2}/(\sigma g^{2}+\sigma ge^{2}/l+\sigma E^{2}/rl)$ and $\sigma E^{2}$ re variance components of genotype, genotype × location, and error residuals, respectively, *l* and *r* are the number of locations and replications, respectively. Karl Pearson’s coefficient of correlation was predicted for all the traits analysed in this study.

### 2.7 Genotype plus genotype-by-environment model on multi-environment factor

Site regression models (commonly known as genotype plus genotype-by-environment (GGE biplot) (Yan and Kang, 2003) were used to visualise the GEI patterns and to understand (1) the which-won-where pattern whereby specific genotypes can be recommended to a specific environment(s), (2) genotype evaluation and stable genotype across all environments, and (3) environment evaluation, which explains discriminative power among genotypes in target environments.

### 2.8 Multi-trait stability analysis

The multi-trait stability index (MTSI) was implemented by Olivoto and Lúcio (2020) in the Metan r package to predict the stability and mean performance across all the traits together. The MTSI was estimated using the WAASBY index based on the superiority index based on mixed-effect models


$$WAASBY_{i}=\frac{\left(rG_{i}x\theta_{y})+(rW_{i}x\theta_{s}\right)}{\theta_{y}+\theta_{s}}$$

(Olivoto et al., 2019).

## 3 Results

### 3.1 Mean performance for agronomic traits

The days required to reach 50% flowering directly influence grain yield and productivity. Therefore, the mean performance for 50% flowering of pearl millet hybrids was recorded in all environments to understand the environmental effects of 50% flowering (**Table 1**). Days to 50% flowering varied from 48.15 to 55.09 days among the 20 pearl millet hybrids (**Supplementary Figure S2A**). Among environments, the mean value for 50% flowering varied from 43.32 (Konni) to 63.15 (Senegal) days. The hybrid ICMX207059 exhibited early flowering in four locations, such as Ghana, Konni, Senegal, and Nigeria, whereas late flowering was observed at Sadore for the ICMX207171 hybrid (**Supplementary Figure S3A**). Pearl millet hybrid ICMX207199 demonstrated late flowering in Senegal, Nigeria, and Ghana, which varied from 46.97 to 56.40 days. The overall maximum days required to reach 50% flowering at Konni varied from 58.67 to 68.18 days at the Konni location (**Supplementary Figure S4**).

**Table 1:** Means of agronomic, starch, and mineral traits for 20 pearl millet hybrids tested across five locations in West Africa.

Entry	Genotype	DF50	PH	PL	GY	Fe	Zn	SDS	RS	TS
1	ICMH177111	53.63	229.18	36.63	2.07	35.35	38.74	48.43	3.49	71.44
2	ICMX207059	48.15	190.10	35.59	1.88	33.71	32.31	47.44	2.30	70.26
3	ICMX207070	53.47	234.80	34.51	1.95	37.33	36.11	47.78	2.70	70.78
4	ICMX207076	54.24	229.17	40.29	1.74	31.08	36.03	47.53	2.99	70.97
5	ICMX207094	52.58	245.33	40.78	2.20	33.89	40.04	46.48	2.15	69.08
6	ICMX207136	51.40	209.37	28.92	1.39	47.54	41.59	47.15	2.16	70.41
7	ICMX207137	51.82	223.05	31.47	2.73	43.62	36.76	47.70	3.17	71.76
8	ICMX207160	54.40	253.61	28.97	3.15	32.67	32.26	47.96	2.81	71.37
9	ICMX207167	51.82	228.83	28.36	2.27	32.76	31.62	46.53	2.84	69.45
10	ICMX207171	53.48	231.27	30.32	1.79	40.89	36.37	46.97	2.33	70.09
11	ICMX207181	54.52	222.65	30.71	2.24	33.04	34.57	47.60	3.24	70.71
12	ICMX207183	54.54	249.19	36.23	2.81	33.04	32.99	47.26	3.06	70.80
13	ICMX207184	52.22	241.81	23.64	2.93	28.17	33.39	47.61	2.73	70.74
14	ICMX207190	50.44	229.40	25.64	2.72	40.01	38.35	47.60	2.99	70.91
15	ICMX207191	54.02	257.08	28.52	2.41	31.32	34.11	47.28	2.59	70.42
16	ICMX207192	50.22	217.51	29.19	2.48	32.38	30.88	46.71	3.58	70.06
17	ICMX207198	52.93	226.48	28.00	2.78	34.01	37.02	47.17	2.47	70.08
18	ICMX207199	55.09	257.54	31.88	1.79	30.83	32.31	46.62	2.74	69.11
19	ICMX207207	53.53	247.52	32.29	2.95	31.91	34.66	47.62	2.67	70.73
20	ICMX207211	48.45	204.20	25.07	2.90	34.75	33.75	46.55	2.63	69.22
Mean		52.55	231.40	31.35	2.36	34.92	35.19	47.30	2.78	70.42
CV%		3.42	4.66	11.78	18.38	9.95	9.56	2.85	20.69	1.83

The average plant heights were recorded for the 20 hybrids to understand the correlation of gain yield with plant height. The average range of plant height varied from 218.66 to 244.64 cm among various environmental conditions (**Supplementary Figure S2A**). The hybrid line ICMX207059 showed minimum average height (190.10 cm), and a maximum plant height was observed in the ICMX207199 genotype (257.54 cm), taking into account all location data. Maximum average plant height was recorded at the Senegal location, and it varied from 207.47 cm (ICMX207059) to 295.66 cm (ICMX207199). The ICMX207059 genotype showed the lowest height among all five locations, where maximum height was observed for the ICMX207059 genotype in Ghana and Senegal. Higher plant length was also observed for ICMX207094 (268.93 cm) at Konni, ICMX207191 (268.85 cm) in Nigeria, and ICMX207207 (252.80 cm) at Sadore (**Supplementary Figure S4**).

The grain yield (GY) varied from 1.53 to 3.00 t/ha in the five environmental conditions (**Table 1**). The highest amount of grain yield was recorded at 3.00 t/ha in Nigeria followed by 2.99 t/ha in Senegal (**Supplementary Figure S2A**). The average mean performance for grain yield was maximum in ICMX207160, whereas minimum grain yield was observed in ICMX207136. The maximum average grain yield was recorded in ICMX207160 (4.78 t/ha) at the Konni location (**Supplementary Figure S3A**). The grain yield varied from 1.07 to 4.78 t/ha among 20 pearl millet hybrids at the Konni location. The ICMX207136 genotype showed a lower grain yield at all five locations, which ranged from 0.95 to 2.38 t/ha. Higher grain yield was observed in all the hybrids at the Senegal location (**Supplementary Figure S4**).

Panicle length (PL) directly correlates with grain productivity and is influenced by various environmental conditions. The panicle length in our study varied from 27.75 to 35.24 cm in the five environmental conditions in Ghana, Konni, Senegal, Nigeria, and Sadore (**Table 1**). Panicle length was highest in ICMX207094 (40.78 cm), whereas the least panicle length was observed in ICMX207184 (23.64 cm) among all the locations. Longer panicle length was noted in the ICMX207094 genotype (44.67 cm) at the Senegal location, followed by 41.41 cm at the Ghana location. The panicle length varied from 37.18 to 44.67 cm in the environments in which they were evaluated. Panicle length was shorter in size in ICMX207184 in all environmental conditions ranging from 20.03 to 27.52 cm (**Supplementary Figure S4**).

### 3.2 Mean variability for starch-related traits

Phenotypic variability was determined for starch traits such as RDS, SDS, RS, and total starch (TS) in 20 pearl millet (*Pennisetum glaucum*) hybrid at five locations (Ghana, Nigeria, Konni, Senegal, and Sadore) (**Table 2**). The TS content among five environmental conditions was distinctly variable and ranged from 69.17% (Ghana) to 72.69% (Sadore) (**Supplementary Figure S2B**). The average RDS content was recorded highest in Ghana (22.13%) at the Konni location, whereas the lowest was recorded at Sadore (18.72%). In contrast to RDS, SDS content ranged from 45.51% to 50.08% among five locations. Similarly, RS content was also found to be greatly varied among locations and ranged from 1.50% to 3.50% (**Supplementary Figure S3B**). The tester line ICMH177111 and hybrid line ICMX207192 showed the lowest RDS content (19.67% and 19.74% respectively), but the highest RDS content was observed in ICMX207136 (21.03%). The highest SDS content was recorded in ICMH177111 (48.43%), followed by 47.96% in ICMX207160. The minimum SDS content (46.48%) was recorded in ICMX207094. Similarly, ICMX207094 showed the lowest RS content (2.15%), and the maximum RS content was recorded in ICMX207192. Maximum TS content of 71.76% was noted in ICMX207094, whereas the lowest TS content of 69.08% was recorded in the ICMX207137 pearl millet hybrid line (**Supplementary Figure S4**).

**Table 2:** Means of starch component traits in parents of selected hybrids.

Genotype	RDS	SDS	RS	TS	Av. Sta.	SDS/ASTA	RS/TS
IP10520	17.54	38.76	4.53	60.83	56.30	68.85	7.45
IP12839	21.14	45.89	1.60	68.63	67.03	68.46	2.34
IP13180	22.47	45.19	1.51	69.16	67.66	66.79	2.18
IP15872	21.26	45.65	1.15	68.06	66.92	68.22	1.69
IP17150	20.33	43.79	1.87	65.99	64.13	68.29	2.83
IP21020	22.04	47.14	2.76	71.94	69.18	68.15	3.83
IP21155	22.34	45.68	1.70	66.96	65.26	65.76	2.54
IP21169	24.26	45.35	1.52	68.22	66.70	63.63	2.23
IP21206	21.92	44.19	1.67	67.78	66.11	66.85	2.46
IP22445	22.85	44.83	2.19	69.26	67.07	65.93	3.17
IP3030	18.2	39.9	11.0	69.1	58.11	68.68	15.90
IP3757	21.35	42.33	1.60	65.28	63.68	66.48	2.45
IP4974	20.98	43.58	2.00	66.56	64.56	67.50	3.01
IP5560	22.18	46.08	2.20	70.46	68.26	67.51	3.12
IP5900	21.28	43.88	1.37	66.53	65.16	67.34	2.06
IP7967	23.12	47.63	1.58	72.34	70.75	67.32	2.19
IP8166	18.02	37.19	1.54	56.75	55.21	67.37	2.71
IP8182	20.63	43.63	2.53	66.79	64.26	67.89	3.79
IP9840	22.15	45.09	2.12	69.36	67.24	67.06	3.05
ICMA177002	23.79	38.10	0.97	62.86	61.89	61.56	1.54
ICMA177003	19.44	40.83	1.65	61.92	60.28	67.74	2.66
ICMA177111	18.97	49.19	2.50	70.66	68.16	72.17	3.54
ICMR08888	22.38	40.91	1.57	64.85	63.28	64.64	2.41

### 3.3 Mean performance for mineral contents

Pearl millet grain contents recorded in 20 hybrids under five test environmental conditions are presented in **Table 1**. Panicle length is directly correlated with grain productivity and is influenced by various environmental conditions. The average iron content was higher in Ghana (43.88 mg kg^−1^) and lower in Sadore (29.34 mg kg^−1^), indicating the environmental effects on Fe accumulations in hybrid (**Supplementary Figure S2C**). Similarly, the average zinc content was higher in Sadore (39.33 mg kg^−1^) and lower in Nigeria (29.34 mg kg^−1^), demonstrating the role of environmental conditions on Zn content modulation in hybrids. The average Fe content was maximum in the ICMX207136 genotype (47.54 mg kg^−1^), whereas the minimum Fe content was recorded in the ICMX207184 genotype (28.17 mg kg^−1^) among all the locations. Similarly, maximum average Fe content was found in ICMX207136 (41.59 mg kg^−1^) whereas minimum Fe content was recorded in ICMX207192 (30.88 mg kg^−1^) among all the five locations (**Supplementary Figure S3C**). The highest Fe content was noted in ICMX207136 at all locations, which ranged from 40.40 mg kg^−1^ (in environment *x*) to 55.98 mg kg^−1^ (in environment *y*). Lowest Fe content was observed in ICMX207184 and highest in ICMX207094, followed by ICMX207136 (**Supplementary Figure S4**).

### 3.4 Statistical analysis for phenotypic variance among different locations

Combined analysis of variance revealed significant genotypic and location effects at *p*< 0.001 for all agronomic traits. The genotypic variance ranges from 0.19 to 287.36 for the agronomic traits measured. The genotypic variance was highest for plant height, followed by panicle length, days to 50% flowering, and grain yield. The interactions between location and genotype effects exhibited significant differences at *p*< 0.001 for all the agronomic traits such as DF50, PH, PL, and GY (**Table 3**). Broad-sense heritability for agronomic traits (DF50, PH, PL, and GY) was high (>0.70) across five environments, confirming a lower degree of effect of replications on the phenotypic trait, including on starch and yield traits (**Supplementary Figures S5A, B**).

**Table 3:** Variance component estimates from combined analysis of variance for agronomy, starch, and nutrient traits in pearl millet hybrid tested across five locations.

Covariance parameter	Variance components									
	DF50	PH	PL	GY	RDS	SDS	RS	TS	Fe	Zn
Location	52.683^**^	113.130^**^	7.389^**^	0.377^**^	1.675^**^	3.886^**^	0.656^**^	2.080^**^	32.613^**^	8.656^**^
Rep (location)	0.484^**^		0.204	0.050^**^	0.144^**^	0.197^*^	0.047^**^	0.390^**^	0.331	
Genotype	3.390^**^	287.360^**^	23.157^**^	0.197^**^	0.135^**^	0.338^**^	0.159^**^	0.606^**^	22.093^**^	7.536^**^
Location*genotype	3.834^**^	198.360^**^		0.335^**^	0.046	0.147	0.104^**^	0.391^*^	6.609^**^	9.448^**^
Residual (Ghana)	2.839	88.235	5.917	0.193	1.134	2.754	0.099	1.589	20.027	10.714
Residual (Konni)	1.419	210.020	21.618	0.151	0.599	2.033	0.375	2.076	11.535	10.054
Residual (Nigeria)	2.232	114.580	22.485	0.318	0.615	2.146	0.263	2.077	7.834	6.906
Residual (Sadore)	6.974	98.402	10.839	0.103	0.720	1.158	0.633	1.001	10.218	17.442
Residual (Senegal)	2.677	70.358	7.332	0.175	0.454	1.001	0.287	1.552	10.724	11.464

* = P < 0.05, ** = P < 0.01.

The analysis of variance revealed significant (Prob<0.001) genotypic variance for starch-related traits such as RDS, SDS, RS, and TS (**Table 3**). Such highly significant genotypic variance observed for starch traits affirms that the environmental conditions are an important factor in modulating the starch contents in pearl millet. The genotypic variance was higher (0.60) for the TS trait, followed by SDS (0.34) and RDS (0.16). The interactions between genotype and locations were found to be significant for RS and TS traits with a *p*-value of<0.001. The results indicated that noncrossover interaction, i.e., the performance of genotypes varies across locations, was the case for starch traits in this study. GEI was found to be nonsignificant for RDS and SDS, exhibiting that the performance of genotype (i.e., genotypes ranking) is similar across the locations. Broad-sense heritability in general was observed higher for starch traits (RDS, SDS, RS, and TS > 0.69) across five environments (**Table 3**). In individual environments, broad-sense heritability for RDS was variable across environments, ranging from 0.19 (in Konni) to 0.60 (in Sadore), and for SDS, it ranged from 0.38 (in Konni) to 0.72 (in Ghana). However, RS and TS showed higher broad-sense heritability compared to RDS and SDS (a 0.76 (TS) to 0.79 (RS). Considering individual environments, heritability was observed highest in Sadore (0.81 for RS) and Ghana (0.88 for TS) (**Supplementary Figures S5A, B**).

A combined analysis of variance showed significant genotypic variance components under different locations for mineral content such as Fe and Zn traits (**Table 3**). Location-specific variances were also found to be significant for Fe and Zn traits at a *p*-value of<0.001. The genotypic variance was higher (22.093) for iron as compared to Zn (7.536). The interactions between genotype and location were found to be significant for both mineral traits. In the current analysis, both traits were found to be strongly heritable (>0.70) in the combined analysis. Compared with the Zn trait, Fe was found to be more heritable across the five locations. Broad-sense heritability for the Fe trait was higher (>0.80) in Konni and Senegal’s environmental conditions, whereas Zn had a higher scale of heritability in Sadore followed by Senegal (**Supplementary Figures S5A, B**). High heritability for the mineral traits revealed that the phenotypic differences observed among hybrids were due to genetic influences (**Supplementary Figures S5A, B**).

Furthermore, we analysed Karl Pearson’s coefficient of correlations among traits. It was found that the SDS, RS, and TS were positively correlated with each other but negatively with RS. No correlation of starch traits with yield was evident, indicating yield is not affected by these component starch traits. This confirms that the best hybrids identified based on starch traits in our earlier study by Yadav et al. (2022) hold high promise for further on-farm testing, release, and promotion to combat type 2 diabetes (**Table 4**).

**Table 4:** Pearson’s coefficient of correlations among 10 traits across five locations in West Africa.

Traits	RDS	SDS	RS	TS	DF50	PH	GY	Fe	Zn
SDS	−0.10								
RS	−0.74^**^	0.37							
TS	0.03	0.91^**^	0.47^*^						
DF50	−0.19	0.27	0.15	0.24					
PH	−0.13	0.07	0.05	0.06	0.79^**^				
GY	−0.11	0.15	0.26	0.22	−0.06	0.27			
Fe	0.55^*^	0.08	−0.20	0.22	−0.25	−0.40	−0.33		
Zn	0.33	0.20	−0.30	0.16	0.04	−0.11	−0.35	0.66^**^	
PL	−0.15	0.12	0.00	0.07	0.31	0.09	−0.44	−0.08	0.24
Scale	−1				0				+1

**, Significant at 1% level; *, Significant at 5% level. * *P* < 0.05; ** *P* < 0.01.

### 3.5 Genotype plus genotype-by-environment biplots

**Figure 1:**
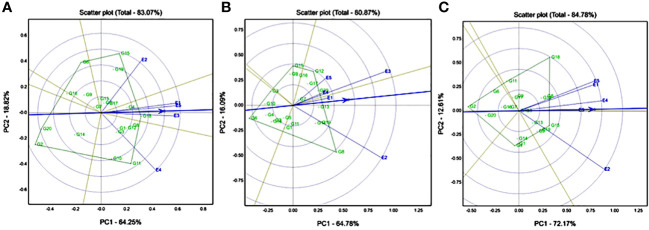
Genotype by genotype × environment (GGE) biplot to identify the favourable environment for **(A)** days to 50% flowering (DF50); **(B)** grain yield (GY); and **(C)** plant height (PH) across the five testing environments.

GGE biplot analysis of multiple traits was used to compare multiple genotypes in multiple environments (five environments) for all the agronomic traits, starch-related traits, and mineral traits (Fe and Zn). GGE-based biplots were generated to link the multiple environmental grades from the origin of the point and found that favourable environmental effects were displayed for agronomic traits, starch-related traits, and mineral traits. The GGE biplot showed quite distinct genotypic variations where the first two interacting principal component axes (IPC) explained 83.07% of the variation for the DF50 trait. According to environmental evaluation, all five locations are highly discriminative, which means genotypic variation is high in each location. E1 (Ghana), E3 (Nigeria), and E5 (Senegal) have formed one mega environment, and they passed through the average environment axis (AEA), confirming they are ideal test environments for days to 50% flowering (DF50). E2 (Konni) and E4 (Sadore) were diverse environments (>90°), i.e., genotype ranks are changing between these two locations. According to genotype evaluation, G15 (ICMX207191) was an adoptable line for E2 (Konni), and G11 (ICMX207181) was an adoptable hybrid line for E4 (Sadore) (**Figure 1A**). Similarly, G18 (ICMX207199) was a high DF50 and stable genotype for all locations. The GGE biplots revealed that E2 (Konni) and E3 are having high discriminating power for yield traits. Genotype G8 (ICMX207160) had a high yield performance at E2 (Konni) location (**Figure 1B**). Similarly, for the plant height trait, E2 (Konni) had a high discriminative location for 20 pearl millet hybrids, and the extensive plant height line was G18 (ICMX207199) (**Figure 1C**).

**Figure 2:**
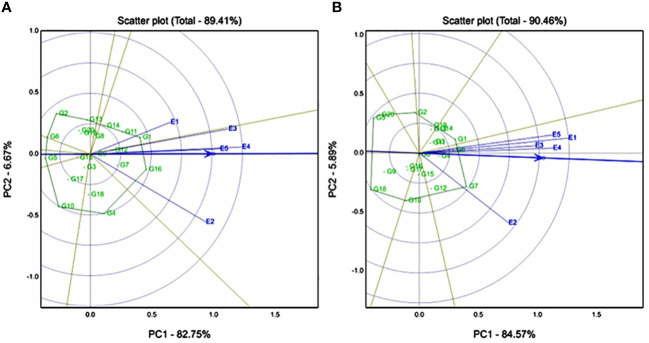
Genotype by genotype × environment (GGE) biplot to identify the favourable environment for **(A)** resistant starch (RS) and **(B)** total starch (TS) across the five testing environments.

Genotype-by-environment interaction was significant for RS and TS, whereas RDS and SDS showed nonsignificant interactions. The first two IPCs explained 89.41%, and 90.46% variation for both resistant and total starch traits, respectively. Biplots showed that E4 (Sadore) had the longest vector and passes through the AEA, an E4 is more ideal test location for the resistant starch trait. However, compared to E2 (Konni), all remaining locations are highly discriminative for a total starch trait. Genotypes G16 (ICMX207192) and G1 (ICMH 177111) were found to be the best performing and stable hybrids for resistant starch trait (**Figure 2A**), whereas G1 (ICMH 177111), G8 (ICMX207160), and G7 (ICMX207137) were the highest performing and stable hybrids for the total starch trait (**Figure 2B**).

**Figure 3:**
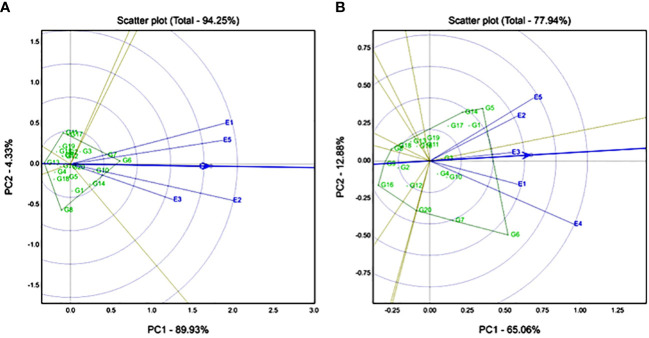
Genotype by genotype × environment (GGE) biplot to identify the favourable environment for **(A)** iron (Fe) and **(B)** zinc (Zn) across the five testing environments.

According to Fe, the GGE biplot explained 94.25% of the variation for the Fe trait (**Figure 3A**). E2 (Konni), E1 (Ghana), and E5 (Senegal) had high discriminative locations compared to E3 (Nigeria) and E4 (Sadore). Genotypes G6 (ICMX207136) and G7 (ICMX207137) had high Fe content and were found stable across all the locations. Accordingly for Zn, the GGE biplot explained 77.94% of the variation for the Zn trait (**Figure 3B**), and E4 (Sadore) and E5 (Senegal) were found to be highly discriminative locations compared with the rest of the locations for Zn trait (**Figure 3B**). G5 (ICMX207094), G14 (ICMX207190), G1 (ICMH177111), and G17 (ICMX207198) are the best adaptable hybrid for E2 (Konni) and E5 (Senegal). Genotype G6 (ICMX207136) was an adoptable hybrid line for E4 (Sadore) and E1 (Ghana) locations.

### 3.6 Best-performing and highly stable entries

**Figure 4:**
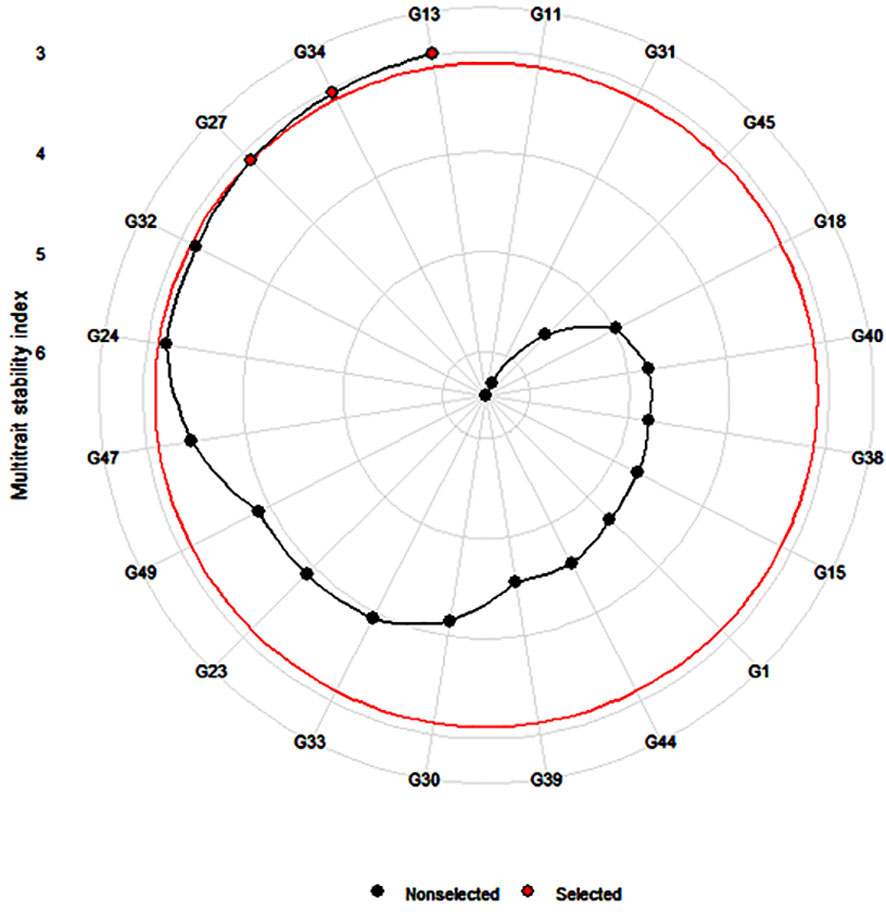
Prediction of highly stable and best-performing genotypes (G13 = ICMX207070, G27 = ICMX207160, and G34 = ICMX207184) across all the traits in pearl millet lines tested at five locations.

MTSI was calculated by estimating the WAASBY index for genotype stability and mean performance across all the traits, such as agronomic (DF50, PH, PL, and GY), starch trait (RDS, SDS, RS, and TS), and mineral (Fe and Zn) traits. The result indicated that genotypes G13 (ICMX207070) and G27 (ICMX207160) and G34 (ICMX207184) were found to be the best performing and stable among the five test environments. The stability index score was lowest for genotype G18 (ICMX207094), showing poor stability and mean performance among all the environmental conditions (**Table 4** and **Figure 4**).

## 4 Discussion

Yield enhancement has long been the goal of plant science research across disciplines, using different genetic and molecular breeding approaches. In recent years, the integration of grain yield with nutritional traits has been better recognised, and this study had such an aim. In this study, we assessed the genetic variation for agronomic, nutritional, and starch traits in selected genotypes identified earlier from within the world collection of pearl millet germplasm based on their starch contents (Yadav et al., 2022). An extremity of genetic variations was observed in the traits measured across five locations due to differences in environmental conditions across these locations. ANOVA analysis demonstrated highly significant differences (*p*< 0.001) for genotypes, environments, and their GEI for yield-associated traits and micronutrient contents, which means genotype ranks are significantly changing across the environments. Similar results were reported by Sankar et al. (2021) for traits such as days to 50% flowering and foliar blast disease of pearl millet in testing environments, as reported in this study. Similar results were also reported in closely related crop finger millet by Adugna et al. (2011); Molla et al. (2013); Dagnachew et al. (2014); Birhanu et al. (2016); Lakew et al. (2017), and Seyoum et al. (2019). However, Dehghani et al. (2006); Tolessa et al. (2013), and Singamsetti et al. (2021) also reported significant interactions in pea, wheat, and maize across environments and genotypes.

In addition to yield parameter, pearl millet grain has several advantages for consumption as it is rich in soluble and insoluble dietary fibres (Ragaee et al., 2006; Kam et al., 2016). Consumption of significant quantities of dietary fibre has been shown to be beneficial to human nutrition, helping reduce the risk of certain types of cancer, coronary heart disease, diabetes, and constipation. Dietary starch is classified into three main fractions based on its *in vitro* digestibility, namely, rapidly digestible starch, slowly digestible starch, and RS. Among these, the RS fraction in the food resists digestion in the small intestine and passes on to the colon. In the colon, RS serves as a substrate for fermentation by the intestinal microbiome to produce short-chain fatty acids. Hence, when RS replaces available carbohydrates in the meal, postprandial glycaemia is reduced, which helps patients, especially those with T2D, normalise their glucose pressure. In this study, the average mean performance of RS content of 20 pearl millet hybrid was assessed in five testing environments and showed a high degree of variations among genotypes (2.15% and 3.58%) as well as among testing environments (1.50% and 3.78%). A previous study reported by Yadav et al. (2022) detected a range of variations in RS content in pearl millet inbred lines, ranging from 0.08% to 1.99%. However, a slightly higher RS content was reported by Zhang et al. (2020) in rice, which ranged from 0% to 3.47%, with an average of 1.67%. In the present study, we have used MTSI and AMMI ASV for the selection of the best hybrids. The main difference between MTSI and AMMI ASV is that the MTSI index is calculated to select lines with amended and consistent performance for multiple traits simultaneously, while the ASI is the rank of genotypes based on their stability index value for each of the traits. The analysis of variance for the RS trait exhibited a significant degree of variations and genotypes and the GEI effects accounted for approximately 89.41% of the total variation. The statistical analysis confirmed that genotypes G16 (ICMX207192) and G1 (ICMH177111) were found to be the most stable and best-performing hybrids for the resistant starch trait among five testing environments such as Ghana, Nigeria, Sadore, Senegal, and Konni. The Sadore environment is a more ideal test environment, which has a high discriminating ability and a representative and identified ranking of genotypes that are not much varied across the environments; meanwhile, the respective genotypes (G16 and G1) are stable and eminently performed genotypes across the environments. These lines of pearl millet can be considered a good source of dietary fibres and have a low glycaemic index with increased levels as compared to other cereals (Sankar et al., 2021). Similarly, SDS are an equally important component of carbohydrates that gets processed in the small intestine and helpful in type 2 diabetes management by releasing the energy slowly. In the present study, the best pearl millet hybrid showing high average mean performance for SDS across five testing environments was identified, which ranged from 46.48% to 46.48%. Interestingly slowly digestible starch was nonsignificant for genotypic and environmental interactions, but it had high broad-sense heritability, indicating the lower environmental influence on its measurement. The actual differences in the five physical distant environments at five locations significantly affected SDS. High levels of SDS within the food are directly associated with a low glycaemic index (GI), and high SDS foods can therefore form an important part of the diet to help mitigate the risk of diverse chronic degenerative diseases such as type 2 diabetes and other obesity-related disorders (Englyst et al., 1996; Parween et al., 2020).

Inadequacies of Fe and Zn micronutrients are considerable health concerns, especially in developing countries with deprived and resource-poor people. In this study, we have estimated the mineral content, such as Fe and Zn, in pearl millet hybrid. The average Fe content was 34.91 mg kg^−1^, and the average Zn was 35.19 mg kg^−1^ in the pearl millet hybrid. Similar results were reported by Govindaraj et al. (2019) for the estimation of mineral content in pearl millet hybrids lines, and it ranged from 25.8 to 80.0 ppm for Fe and 22.0 to 70 ppm for Zn. They have also reported the mineral content in commercial pearl millet hybrids, which ranged from 31.0 to 61.0 ppm for Fe and 32.0 to 54.0 ppm for Zn. An earlier report by Mahendrakar et al. (2019) concluded that pearl millet grains contain Fe (22–154 ppm) and Zn (19–121), which are relatively higher amounts of the Fe and Zn. Analysis of 281 advanced breeding lines exhibited substantial variability for Fe (35–116 ppm) and Zn (21–80 ppm) (Pujar et al., 2020a). ICTP8203, an open-pollinated variety (OPV) released in India in 1988, had been found to have the highest level of Fe and Zn density (Rai et al., 2013). In the current study, we also dissected the relationship of grain yield with micronutrients and starch contents, which was found neutral, indicating that micronutrients and starch can be improved without any penalty ion yield per se.

## 5 Conclusion

In the present study, we report a significant level of phenotypic variability for agronomic traits among the entries selected to possess high concentrations of SDS and RS in their grains. Starch traits in hybrids of these entries did not show any correlation to grain yields across five distinct locations in West Africa, confirming that starch traits can be improved without any penalty on grain yield. Genotype-by-environment interaction studies of these hybrids identified no GEI interactions on starch traits, but the locations/environment significantly affected the starch traits, especially the slowly digestible starch. GGE biplots depicted environment-specific adaptability and identified the winning genotypes for the five testing environments. Among the tested genotypes, G13 (ICMX207070), G27 (ICMX207160), and G34 (ICMX207184) were identified as the most stable and best-performing hybrid among the five testing environments.

## Data availability statement

The original contributions presented in the study are included in the article/**Supplementary Material**. Further inquiries can be directed to the corresponding author.

## Author contributions

RSY and PG conceived the concept of the study. PG helped in designing the hybrid trials, data analysis and curation. CBY, PG, LM and RSY drafted the manuscript. MR, PA, and IA conducted the field trials. All authors read and approved the submitted version.

## References

[B1] Adugna A. Tesso T. Degu E. Tadesse T. Merga F. Legesse F.. (2011). Genotype-by-environment interaction and yield stability analysis in finger millet (Eleucine coracana lGaertn) in Ethiopia. Am. J. Plant Sci. 2, 408–415. doi: 10.4236/ajps.2011.23046

[B2] Antony U. Chandra T. S. (1998). Antinutrient reduction and enhancement in protein, starch and mineral availability in fermented flour of finger millet *(Eleusine coracana*). J. Agric. Food Chem. 46, 2578–2582. doi: 10.1021/jf9706639

[B3] Bidinger F. R. Hash C. T. (2004). “Pearl millet,” in Physiology and biotechnology integration for plant breeding. Eds. Nguyen H. T. Blum A. (New York: Marcel Dekker), pp 225–pp 270.

[B4] Birhanu M. Tesfay M. Nigus C. Wolday K. (2016). Stability analysis of finger millet genotypes in moisture stressed areas of northern Ethiopia. J. Nat. Sci. Res. 2016 (6), 2016.

[B5] Dagnachew L. Masresha F. de Villiers S. Tesfaye K. (2014). Additive main effects and multiplicative interactions (AMMI) and genotype by environment interaction (GGE) biplot analyses aid selection of high yielding and adapted finger millet varieties. J. Appl. Biosci. 76, 6291–6303.

[B6] Dehghani H. Ebadi A. Yousefi A. (2006). Biplot analysis of genotype by environment interaction for barley yield in Iran. J. Agron. 98, 388–393. doi: 10.2134/agronj2004.0310

[B7] Englyst K. N. Englyst H. N. Hudson G. J. Cole T. J. Cummings J. H. (1999). Rapidly available glucose in foods: an *in vitro* measurement that reflects the glycaemic response. Am. J. Clin. Nutr. 69, 448–454. doi: 10.1093/ajcn/69.3.448 10075329

[B8] Englyst H. N. Kingman S. M. Cummings J. H. (1992). Classification and measurement of nutritionally important starch fractions. Eur. J. Clin. Nutr. 46, S33–S50. 1330528

[B9] Englyst H. N. Kingman S. M. Hudson G. J. Cummings J. H. (1996). Measurement of resistant starch *in vitro* and *in vivo. Br* . J. Nutr. 75, 749–755. doi: 10.1079/BJN19960178 8695601

[B10] Govindaraj M. Rai K. N. Cherian B. Pfeiffer W. H. Kanatti A. Shivade H. (2019). Breeding biofortified pearl millet varieties and hybrids to enhance millet markets for human nutrition. Agriculture 9 (5), 106. doi: 10.3390/agriculture9050106

[B11] Govindaraj M. Shanmugasundaram P. Sumathi P. Muthiah A. R. (2010). Simple, rapid and cost-effective screening method for drought resistant breeding in pearl millet. Elec. J. Plant Breed. 1, 590–599.

[B12] Guild G. E. Stangoulis J. C. R. (2016). Non-matrix matched glass disk calibration standards improve XRF micronutrient analysis of wheat grain across five laboratories in India. Front. Plant Sci. 7, 147–110. doi: 10.3389/fpls.2016.00784 27375644 PMC4896964

[B13] Gupta S. K. Rai K. N. Singh P. Ameta V. L. Gupta S. K. Jayalekha A. K.. (2015). Seed set variability under high temperatures during flowering period in pearl millet (Pennisetum glaucum l. (R.) br.). Field Crops Res. 171, 41–53. doi: 10.1016/j.fcr.2014.11.005

[B14] Institute Inc S. A. S. (2018). SAS/STAT® 15.1 user’s guide (Cary, NC: SAS Institute Inc).

[B15] Kam J. Puranik S. Yadav R. Manwaring H. R. Pierre S. Srivastava R. K.. (2016). Dietary interventions for type 2 diabetes: how millet comes to help. Front. Plant Scie. 7 14, 01454. doi: 10.3389/fpls.2016.01454 PMC5037128 27729921

[B16] Lakew T. Dessie A. Tariku S. Abebe D. (2017). Evaluation of performance and yield stability analysis based on AMMI and GGE models in introduced upland rice genotypes tested across Northwest Ethiopia. Int. J. Res. Stud. Agric. Sci. 3, 17–24.

[B17] Mahendrakar M. D. Kumar S. Singh R. B. Rathore A. Potupureddi G. Kavi Kishor P. B.. (2019). Genetic variability, genotype × environment interaction and correlation analysis for grain iron and zinc contents in recombinant inbred line population of pearl millet [*Pennisetum glaucum* (L). r. br.]. Indian J. Genet. 79 (3), 545–551. doi: 10.31742/IJGPB.79.3.3

[B18] Molla F. Alemayehu A. Belete K. (2013). AMMI analysis of yield performance and stability of finger millet genotypes across different environments. World J. Agric. Res. 9, 231–237.

[B19] Olivoto T. Lúcio A. D. (2020). Metan: an r package for multi-environment trial analysis. Methods Ecol. Evol. 11, 783–789. doi: 10.1111/2041-210X.13384

[B20] Olivoto T. Lucio A. D. C. Silva J. A. G. Marchioro V. S. de Souza V. Q. Jost E.. (2019). Mean performance and stability in multi-environment trials I: combining features of AMMI and BLUP techniques. Agron. J. 111, 2949–2960. doi: 10.2134/agronj2019.03.0220

[B21] Paltridge N. G. Milham P. J. Ortiz-Monasterio J. I.. (2012a). Energy-dispersive X-ray fluorescence spectrometry as a tool for zinc, iron and selenium analysis in whole grain wheat. Plant Soil 361, 261–269. doi: 10.1007/s11104-012-1423-0

[B22] Paltridge N. G. Palmer L. J. Milham P. J. Guild G. E. Stangoulis J. C. R. (2012b). Energy-dispersive X-ray fluorescence analysis of zinc and iron concentration in rice and pearl millet grain. Plant Soil. 361 (1–2), 251–260. doi: 10.1007/s11104-011-1104-4

[B23] Parween S. Anonuevo J. J. Butardo V. M. Misra G. Anacleto R. Llorente C.. (2020). Balancing the double-edged sword effect of increased resistant starch content and its impact on rice texture: its genetics and molecular physiological mechanisms. Plant Biotech. J. 18, 1763–1777. doi: 10.1111/pbi.13339 PMC7336377 31945237

[B24] Pujar M. Govindaraj M. Gangaprasad S. Kanatti A. Shivade H. (2020). Genetic variation and diversity for grain iron, zinc, protein and agronomic traits in advanced breeding lines of pearl millet [*Pennisetum glaucum* (L.) r. br.] for biofortification breeding. Genet. Resour. Crop Evol., 67, 2009–2022. doi: 10.1007/s10722-020-00956-x

[B25] Ragaee S. Abdelaal E. Noaman M. (2006). Antioxidant activity and nutrient composition of selected cereals for food use. Food Chem. 98 (1), 32–38. doi: 10.1016/j.foodchem.2005.04.039

[B26] Raheem D. Dayoub M. Birech R. Nakiyemba A. (2021). The contribution of cereal grains to food security and sustainability in Africa: potential application of UAV in Ghana, Nigeria, Uganda, and Namibia. Urban Sci. 5, 8. doi: 10.3390/urbansci5010008

[B27] Rai K. N. Yadav O. P. Rajpurohit B. S. Patil H. T. Govindaraj M. Khairwal I. S.. (2013). Breeding pearl millet cultivars for high iron density with zinc density as an associated trait. J. SAT Agri. Res. 11, 1–7.

[B28] Sankar S. M. Singh S. P. Prakash G. Satyavathi C. T. Soumya S. L. Yadav Y.. (2021). Deciphering genotype-by- environment interaction for target environmental delineation and identification of stable resistant sources against foliar blast disease of pearl millet. Front. Plant Sci. 12, 656158. doi: 10.3389/fpls.2021.656158 34079568 PMC8165241

[B29] Seyoum A. Semahegn Z. Nega A. Gebreyohannes A. (2019). AMMI and GGE analysis of GxE and yield stability of finger millet [*Eleusine coracana* (L.) gaertn] genotypes in Ethiopia. Int. J. Trend Res. 6, 379–386.

[B30] Singamsetti A. Shahi J. P. Zaidi P. H. Seetharam K. Vinayan M. T. Kumar M.. (2021). Genotype x environment interaction and selection of maize (*Zea mays* l.) hybrids across moisture regimes. Field Crops Res. 270, 108224. doi: 10.1016/j.fcr.2021.108224

[B31] Tolessa T. T. Keneni G. Sefera T. Jarso M. Bekele Y. (2013). Genotype by environment interaction and performance stability for grain yield in field pea (*Pisum sativum* l.) genotypes. Int. J. Plant Breed. 7, 116–123.

[B32] Vadivoo A. S. Joseph R. Ganesan N. M. (1998). Genetic variability and diversity for protein and calcium contents in finger millet (*Eleusine coracana* (L) gaertn) in relation to grain colour. Plant Foods Hum. Nutr. 52, 353–364. doi: 10.1023/A:1008074002390 10426122

[B33] Yadav S. L. Beniwal B. R. Rajpurohit B. S. Kumar M. Yadav H. P. (2015). Response of genotypes to heat tolerance at 42°C in pearl millet [*Pennisetum glaucum* (L.) r. br.]. Environ. Ecol. 33, 341–344.

[B34] Yadav R. Hash C. T. Bidinger F. R. Cavan G. P. Howarth C. (2002). Quantitative trait loci associated with traits determining grain and stover yield in pearl millet under terminal drought stress conditions. Theor. Appl. Genet. 104 (1), 67–83. doi: 10.1007/s001220200008 12579430

[B35] Yadav C. B. Srivastava R. K. Beynon S. Englyst K. Gangashetty P. I. Yadav R. S. (2022). Genetic variability and genome-wide marker association studies for starch traits contributing to low glycaemic index in pearl millet. Food Energy Secur. 11, e341. doi: 10.1002/fes3.341

[B36] Yan W. Kang M. S. (2003). GGE biplot analysis: a graphical tool for breeders, geneticists, and agronomists (Boca Raton, FL: CRC Press).

[B37] Zhang N. Wang M. Fu J. Shen Y. Ding Y. Wu D.. (2020). Identifying genes for resistant starch, slowly digestible starch, and rapidly digestible starch in rice using genome-wide association studies. Genes Genomics 42, 1227–1238. doi: 10.1007/s13258-020-00981-1 32901332

