# Supplementary material for: Genotype-by-environment interactions for starch, mineral, and agronomic traits in pearl millet hybrids evaluated across five locations in West Africa
Source: Front Plant Sci. 2023 May 23;14:1171773. doi: 10.3389/fpls.2023.1171773 (PMC10242008; doi:10.3389/fpls.2023.1171773)
Supplement: Supplementary file 1 [file Presentation_1.pptx]

## Slide 1
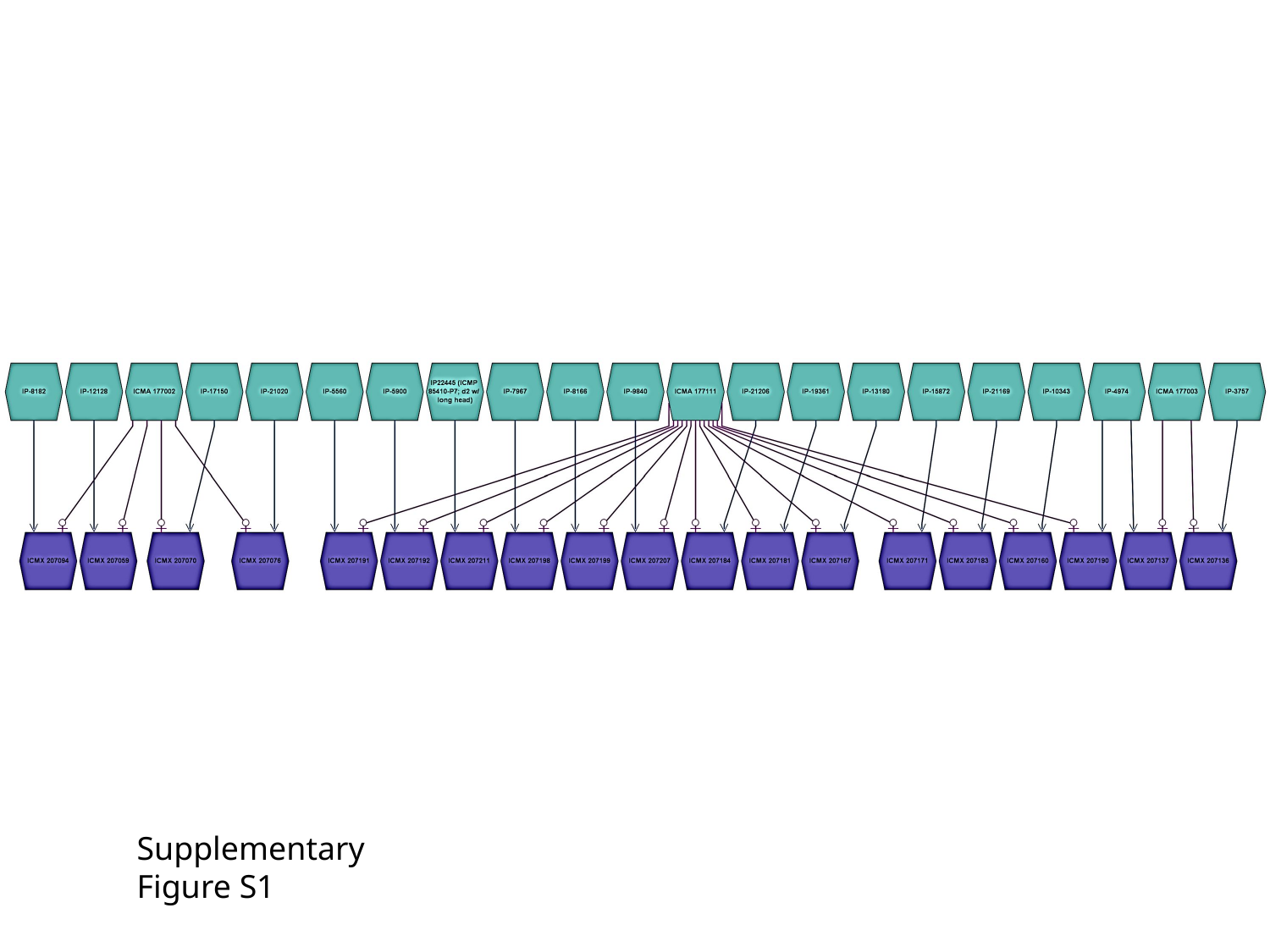

Supplementary Figure S1

## Slide 2
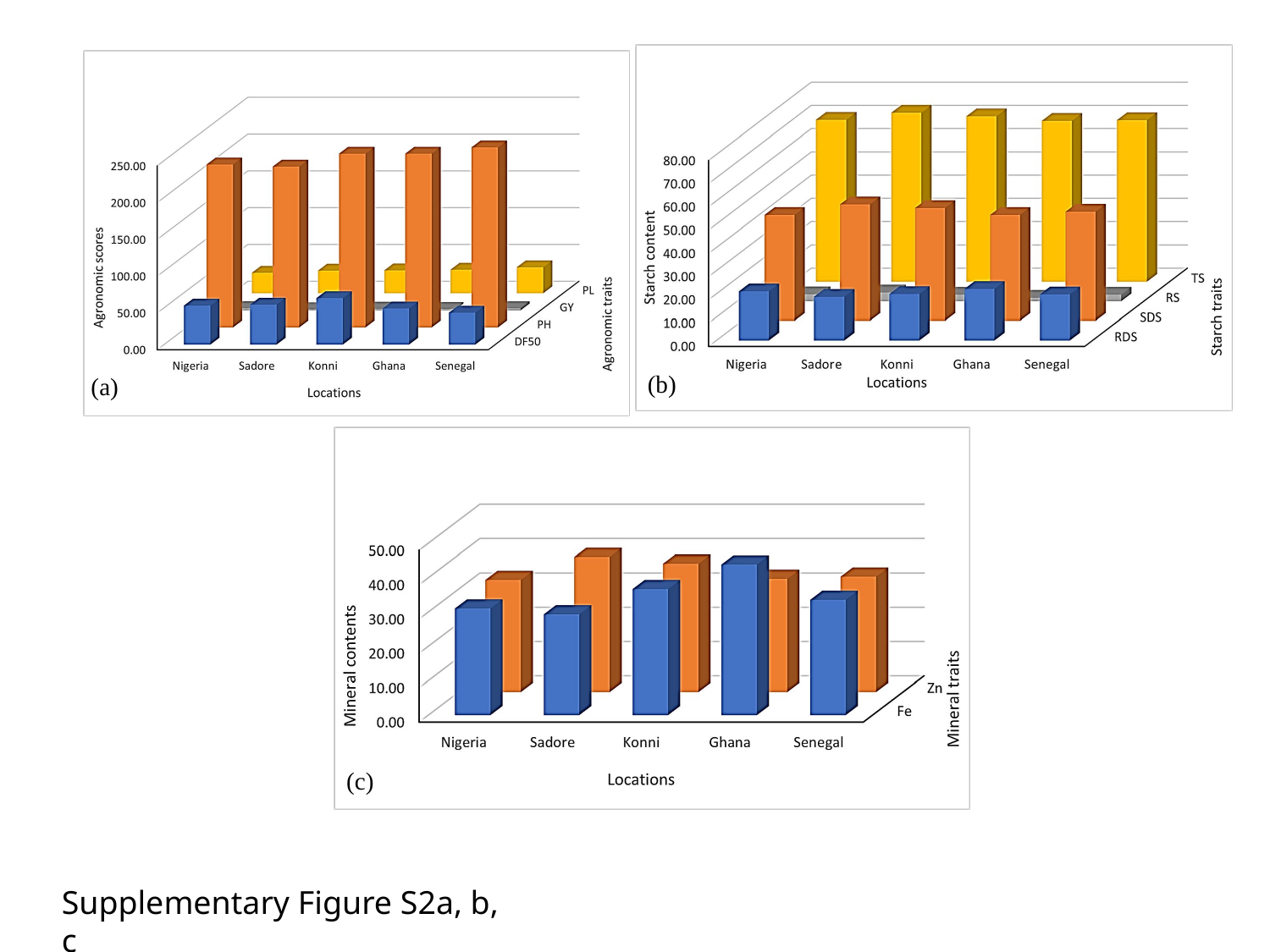

(b)
(a)
(c)
Supplementary Figure S2a, b, c

## Slide 3
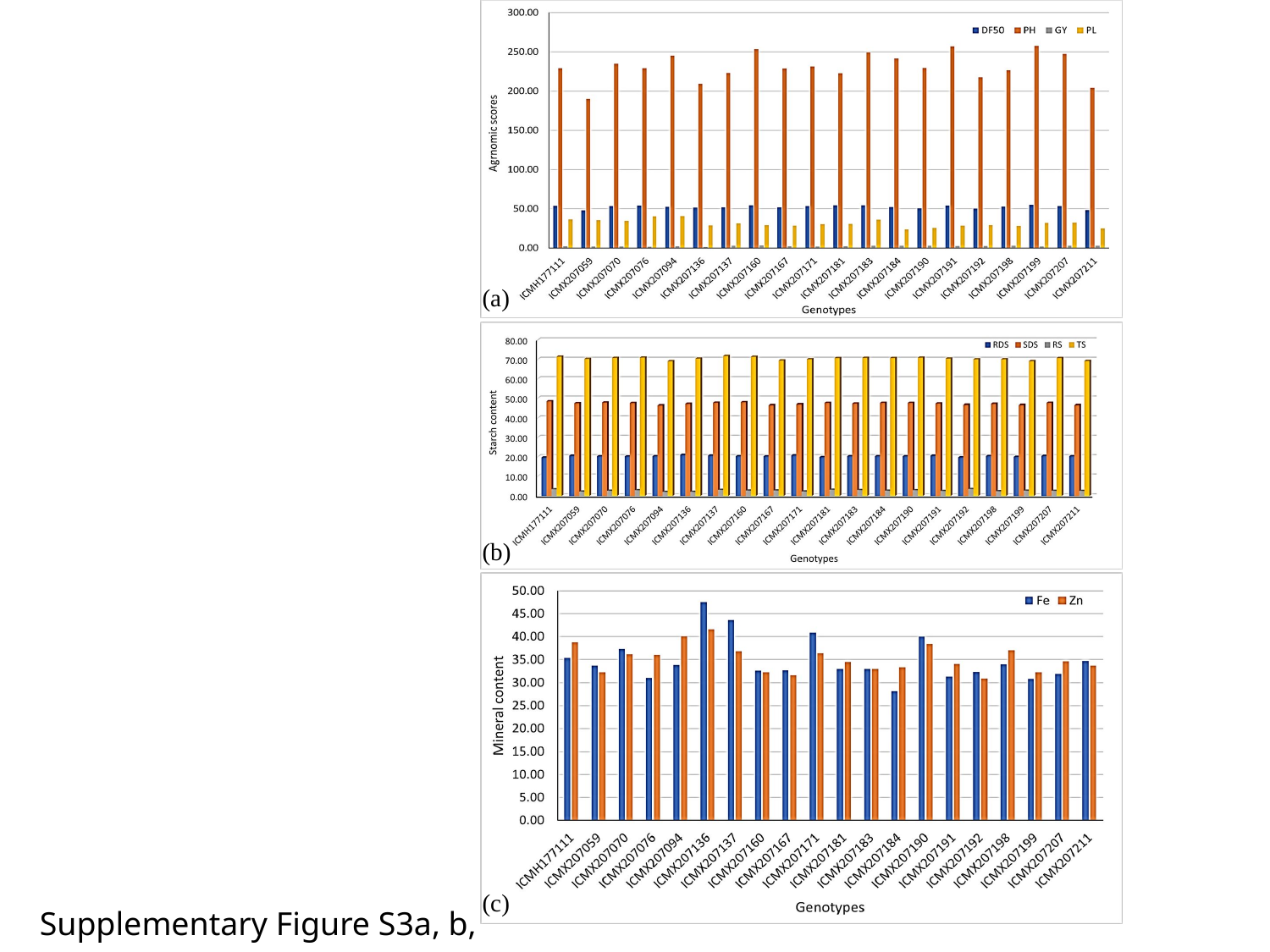

(a)
(b)
(c)
Supplementary Figure S3a, b, c

## Slide 4
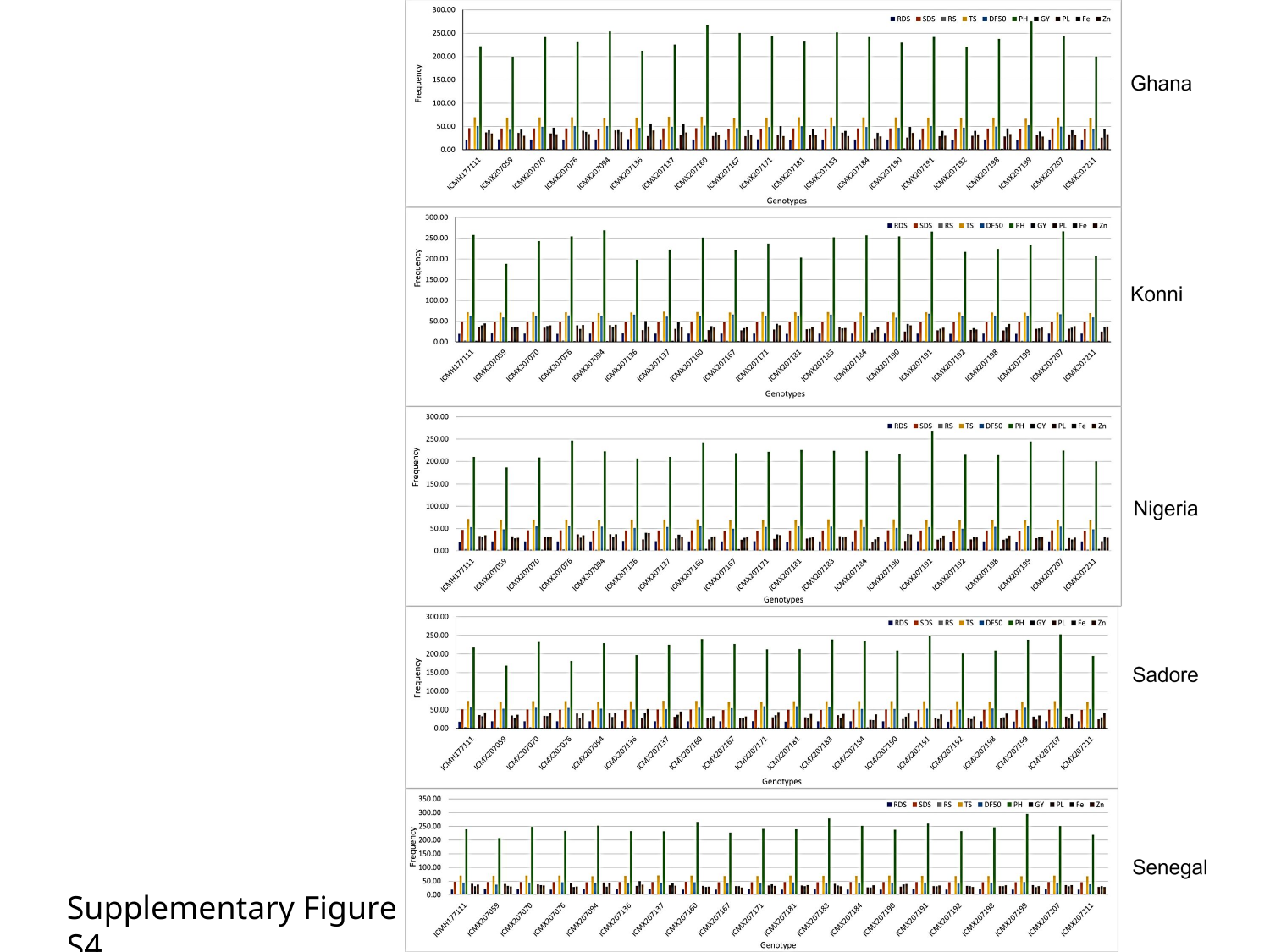

Supplementary Figure S4

## Slide 5
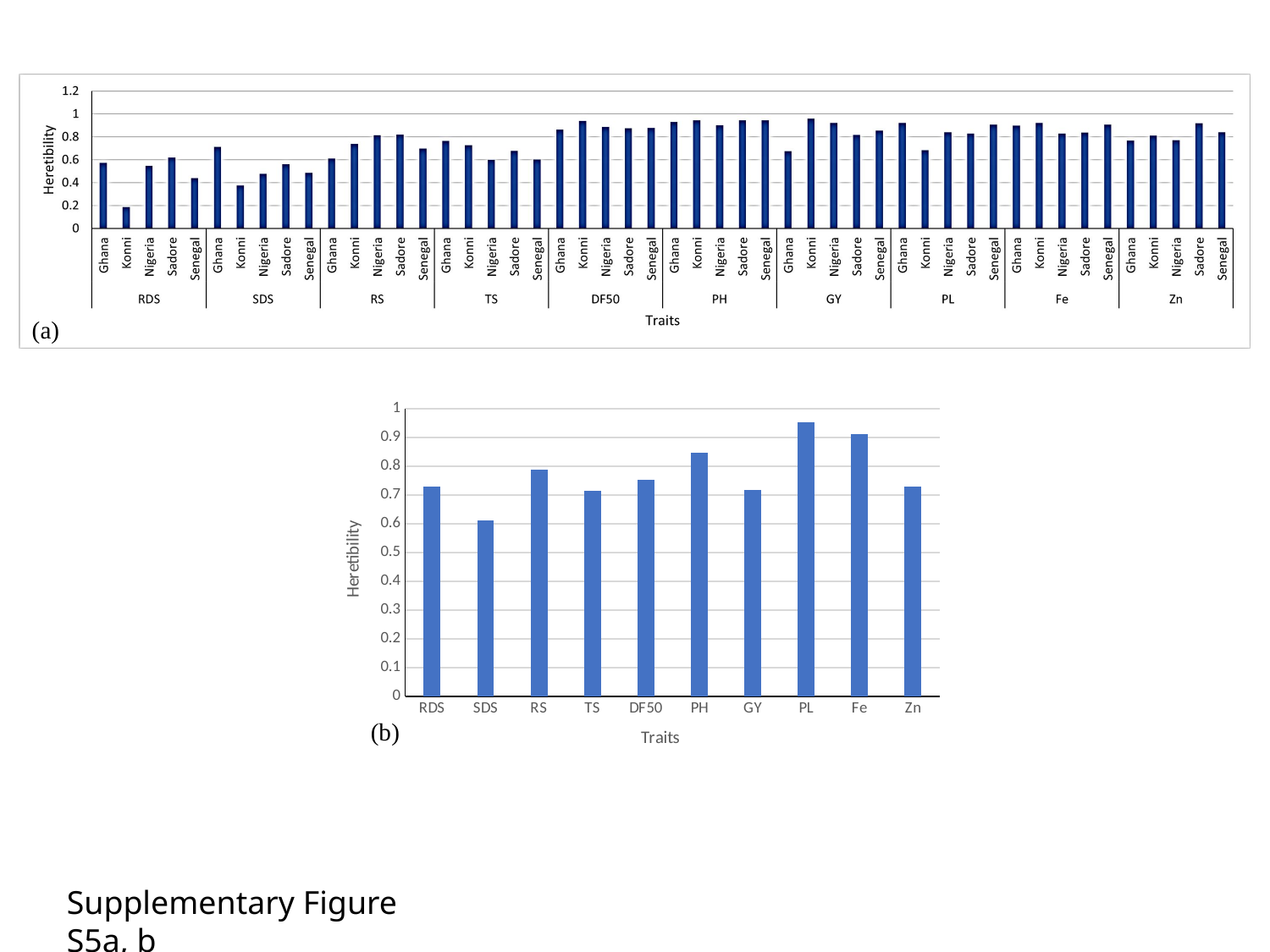

(a)
### Chart
| Category | |
|---|---|
| RDS | 0.730446064492229 |
| SDS | 0.612654523854028 |
| RS | 0.788465482888311 |
| TS | 0.714221918135255 |
| DF50 | 0.752972122954552 |
| PH | 0.845824749247206 |
| GY | 0.716181289873157 |
| PL | 0.952087710152696 |
| Fe | 0.911963929523444 |
| Zn | 0.728872551644316 |(b)
Supplementary Figure S5a, b
